# Infrared Hollow Optical Fiber Probe for Localized Carbon Dioxide Measurement in Respiratory Tracts

**DOI:** 10.3390/s18040995

**Published:** 2018-03-27

**Authors:** Takashi Katagiri, Kyosuke Shibayama, Takeru Iida, Yuji Matsuura

**Affiliations:** 1 Graduate School of Engineering, Tohoku University, 6-6-05 Aoba, Sendai 980-8579, Japan; katagiri@ecei.tohoku.ac.jp; 2 Graduate School of Biomedical Engineering, Tohoku University, 6-6-05 Aoba, Sendai 980-8579, Japan; kyosuke.shibayama.p2@dc.tohoku.ac.jp (K.S.); ru_take@ecei.tohoku.ac.jp (T.I.)

**Keywords:** infrared absorption spectroscopy, carbon dioxide measurement, functional bronchoscopy, optical fiber sensor

## Abstract

A real-time gas monitoring system based on optical absorption spectroscopy is proposed for localized carbon dioxide (CO_2_) measurement in respiratory tracts. In this system, a small gas cell is attached to the end of a hollow optical fiber that delivers mid-infrared light with small transmission loss. The diameters of the fiber and the gas cell are smaller than 1.2 mm so that the probe can be inserted into a working channel of common bronchoscopes. The dimensions of the gas cell are designed based on absorption spectra of CO_2_ standard gases in the 4.2 μm wavelength region, which are measured using a Fourier-transform infrared spectrometer. A miniature gas cell that is comprised of a stainless-steel tube with slots for gas inlet and a micro-mirror is fabricated. A compact probing system with a quantum cascade laser (QCL) light source is built using a gas cell with a hollow optical fiber for monitoring CO_2_ concentration. Experimental results using human breaths show the feasibility of the system for in-situ measurement of localized CO_2_ concentration in human airways.

## 1. Introduction

Imaging lung functions is essential for planning procedures in surgery and the treatment of pulmonary emphysema and atelectasis [1,2]. A computed tomography (CT) is the most commonly used method for functional lung imaging that provides three-dimensional high-resolution images [3]. However, the radiation dose for the X-ray-based methods represented by CT imaging is always of great concern to patients when applied in vivo. Lung ventilation scintigraphy based on images of radioactive gases taken by a gamma camera is also commonly used to visualize localized ventilation functions of lungs [4]. However, the use of radioactive materials and low image resolution are problematic with this method.

Aside from these methods that provide information on tissue configuration and volume, direct measurement of localized gas concentration and airflow using a catheter provides more detailed information on localized lung functions [5,6,7,8]. Mantri et al. developed a system for measuring collateral ventilation [5]. A balloon inserted through a working channel of a bronchoscope was inflated to isolate the targeted lung compartment and measure airway resistance. Yoshimasu et al. used a common capnometer to measure localized CO_2_ concentration [6]. A sampling tube was inserted into the orifice of each lobar bronchus to continuously collect inspired and expired gases. Freitag et al. developed a bronchoscope with a catheter in the working channel, which was connected to a suction pump with ventilation valves [7]. It collected gas from airways and used a capnometer and an oximeter to measure the concentration of carbon dioxide and oxygen. The proposed system enables real-time measurement of gas exchange functions and shows real-time concentrations on the same display as bronchoscopic images. However, the suction process causes a time lag in these methods and they are not truly in-situ measurements.

In this paper, we propose a real-time CO_2_ monitoring system that is based on optical absorption spectroscopy. In this system, a small gas cell attached to the end of an optical fiber is inserted into airways through a bronchoscope, thereby enabling in-situ measurements of localized lung functions. The system’s concept and the results of feasibility tests are reported. Recently, a variety of optical-fiber-based gas sensors, such as one using long-period fiber gratings [9,10] and optical fiber-based Fabry Perot interferometer [11]. Unlike these sensors using near infrared light, the system proposed in this paper uses mid-infrared light to directly detect molecular absorption of CO_2_ gas. The simple structure of the proposed fiber-based sensor provides a robust and low-cost gas sensing system. 

## 2. Materials and Methods

**Figure 1:**
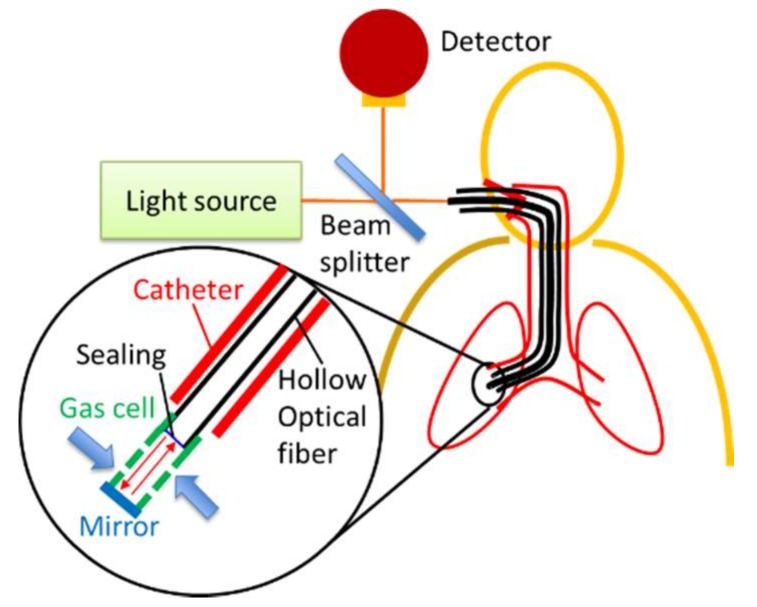
Schematic of proposed fiber-optic probe system for localized gas analysis in a respiratory tract.

Figure 1 schematically shows the gas analysis system based on the optical spectroscopy we propose for real-time analysis of localized lung functions. A small gas cell is attached to the distal end of a thin optical fiber probe that is inserted into airways via a catheter or a bronchoscope. The target gas surrounding the cell penetrates the cell though the small holes or slits on the gas cell. Light delivered by the fiber is introduced into the cell and reflected by the mirror set at the end of the gas cell. The reflected light goes back to the fiber and is detected by an optical detector put outside the body. We used mid-infrared light with a wavelength of around 4.2 μm that is strongly absorbed by CO_2_ gas. However, this system can also be applied to Raman spectroscopy by using a near-infrared laser and a Raman spectroscope. As an optical fiber, we used a thin and flexible hollow optical fiber that shows low transmission loss for mid-infrared light that cannot be delivered by conventional silica-glass optical fibers [12,13,14]. The hollow optical fiber can also be used for Raman spectroscopy because the air in the hollow core does not induce Raman scattering that causes background noise [15]. When using the hollow optical fiber, the distal end of the fiber should be sealed to keep the hollow core away from the gases outside.

**Figure 2:**
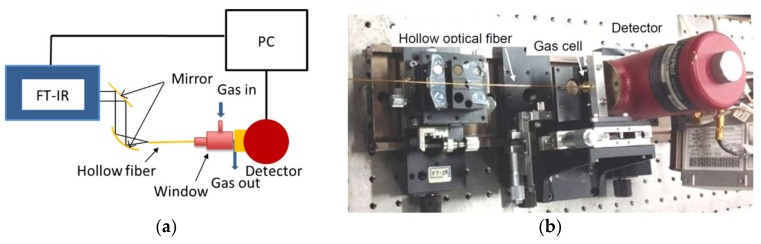
(**a**) Schematic and (**b**) appearance of FT-IR-based gas analysis system used for measuring absorption coefficient of CO_2_ gas.

First, we measured the attenuation coefficient of CO_2_ gas by using the system shown in Figure 2, which is based on Fourier-transform infrared spectroscopy (FT-IR). Infrared light from the FT-IR spectroscope was focused on the input end of a hollow optical fiber with an inner diameter of 700 μm and a length of 10 cm. A small gas cell sealed at both ends with sapphire windows was set at the output end of the fiber and a sample gas was introduced into the cell via the nozzle on the cell. The diameter and the length of the gas cell were 10 mm and 5 mm, respectively. In the experiment, the resolution was set to 2 cm^−1^ and the obtained spectra were integrated 128 times to suppress noises.

**Figure 3:**
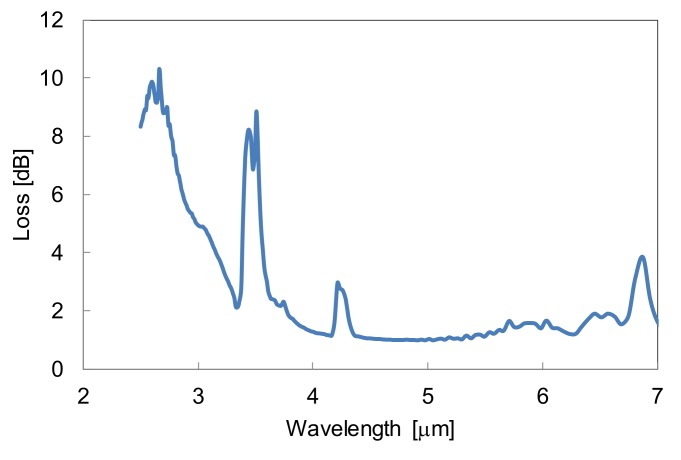
An attenuation loss spectrum of hollow optical fiber with an inner cyclic olefin polymer (COP) film. The inner diameter of the fiber is 700 μm and the length is 120 cm.

The hollow optical fiber that was fabricated in our laboratory was composed of a silica glass capillary tube with a silver thin film inner coating deposited by the silver mirror-plating technique. The top of the silver film is coated with a thin film of cyclic olefin polymer (COP) [16,17]. The polymer layer functions as a dielectric interference coating that enhances the reflection of a targeted wavelength region when the thickness meets the interference conditions. As a result, the transmission loss of the fiber is much lower than the one without a dielectric layer at the targeted wavelength region. Figure 3 shows a loss spectrum of the hollow optical fiber with a COP inner layer used in the CO_2_ measurement system. The small peak at 4.2 μm is from CO_2_ contained in the air core of the fiber, and some absorption peaks of COP appear in the 3.5 μm and 6.5 μm wavelength regions. However, we confirmed that there was no absorption peak of COP around the 4.2 μm region. The large peak at around 2.6 μm is due to the interference effects of the 0.45-μm-thin COP film. With this thickness, a low-loss region appeared around 4.2 μm, where there were absorption peaks of CO_2_.

**Figure 4:**
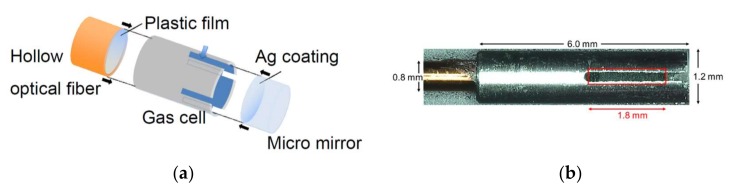
(**a**) Schematic structure and (**b**) appearance of fabricated miniature gas cell. The hollow optical fiber is on the left of the picture.

For clinical applications, we designed and fabricated a miniature gas cell that was attached to the distal end of hollow optical fibers. Figure 4a shows a schematic structure and Figure 4b shows the appearance of the gas cell. A polyvinylidene chloride film was attached to the distal end of the hollow optical fiber by thermocompression bonding to seal the opened distal end. The gas cell was made of a stainless-steel pipe (1.2 mm diameter, 6.0 mm length) that capped the sealed fiber’s end. Four small slits were made on the pipe for introducing surrounding gas into the gas cell. A micro-mirror with a diameter of 1.0 mm was set at the end of the gas cell to reflect light back to the hollow optical fiber. The gap between the sealed fiber end and the mirror was set to 1.8 mm to obtain a good signal-to-noise ratio for the CO_2_ concentrations in breath.

**Figure 5:**
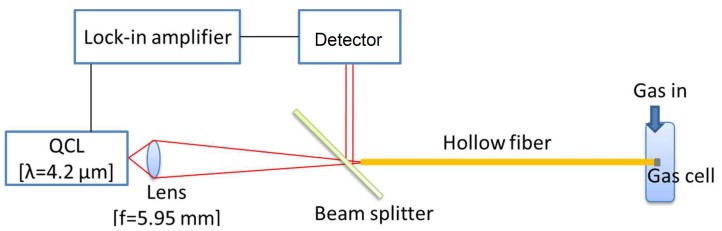
Schematic of the quantum cascade laser (QCL)-based CO_2_ gas analysis system using a hollow optical fiber probe.

Using the miniature gas cell, we built a measurement setup for CO_2_ gas measurement (shown in Figure 5). We used a distributed feedback (DFB) quantum cascade laser (QCL, L12014, Hamamatsu Photonics, Hamamatsu, Japan) as a light source to make the system compact and inexpensive. The wavelength was tuned to around 4.23 μm to coincide with an absorption peak of CO_2_, and the laser emitted pulsed light with a peak power of around 50 mW and a pulse width of 10 ns at a repetition rate of 150 kHz. The temperature of QCL was kept stable by a Peltier element. The laser light was focused on the input end of a hollow optical fiber with a ZnSe aspheric lens with a focal length of 5.95 mm. We used a hollow optical fiber with an inner diameter of 700 μm and a length of 50 cm. The distal end capped with the gas cell was inserted into a plastic bag where a sample gas was injected. The volume of the sample bag was 200 mL, and 100 mL of N_2_ gas was injected into the empty bag for background measurement before the sample gas measurement. In the sample gas measurement, extra gases blew out of a small vent hole in the bag. The laser light that shone back into the fiber was reflected by the beam splitter that was a CaF_2_ plate with a coating providing the beamsplitting ratio of 50:50 at the 4.2-μm band. The reflected beam irradiated an InSb detector (P5968, Hamamatsu Photonics, Hamamatsu, Japan) cooled with liquid nitrogen. A lock-in amplifier (LI5640, NF, Yokohama, Japan) synchronized with the QCL pulses amplified the detected signal.

## 3. Results and Discussion

**Figure 6:**
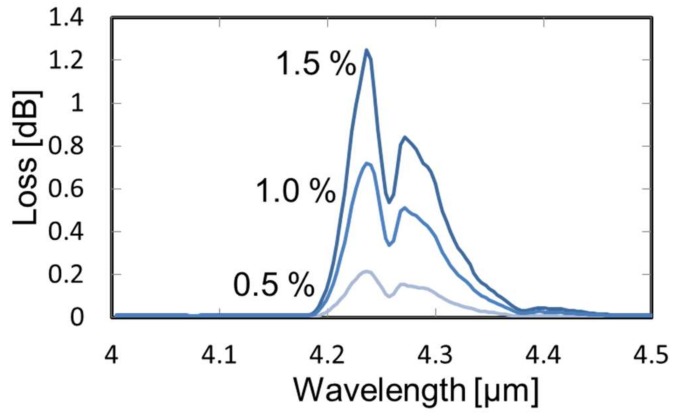
Absorption loss spectra of CO_2_ gas with different concentrations measured by the FT-IR-based gas analysis system.

Figure 6 shows the absorption spectra of CO_2_ gas with different concentrations measured by the FT-IR-based system shown in Figure 2. Two absorption peaks, 4.23 μm and 4.28 μm, were clearly observed, and the peak intensities changed with the concentration. More than five measurements were performed for each concentration, and deviation in the measured absorption loss was ±0.05 dB, which corresponded to ±0.05% of concentration. We set the target wavelength for CO_2_ detection as 4.23 μm based on these results.

**Figure 7:**
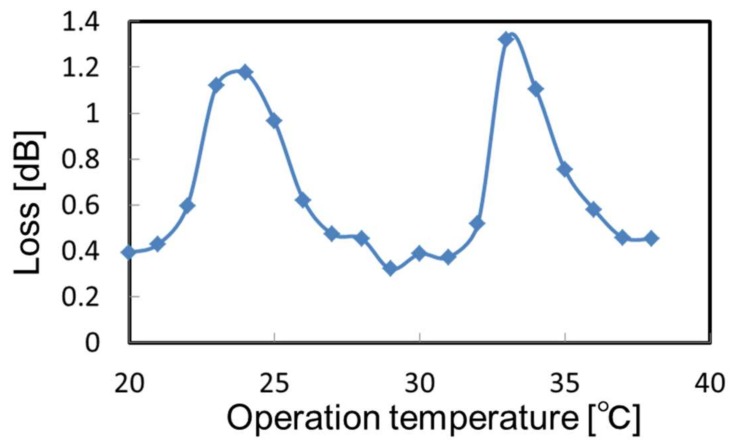
Change in the absorption loss of 4% CO_2_ gas measured by a QCL with different operating temperatures.

We tuned the emitting wavelength of the laser using the QCL-based system shown in Figure 5. The line width of the emitting wavelength was less than 0.2 cm^−1^ and could be shifted about 2.0 cm^−1^ by changing the operation temperature from −10 to 50 °C. In this test, the sample bag was filled with 4% CO_2_ gas and the emitting wavelength was changed by increasing the operating temperature of the laser. Figure 7 shows the change in the measured absorption loss. One can see sharp absorption peaks of different vibration rotational levels that were not seen in the spectra measured by the FT-IR system with a much lower resolution than the QCL line width (shown in Figure 6). We set the operational temperature to 24 °C based on this result.

**Figure 8:**
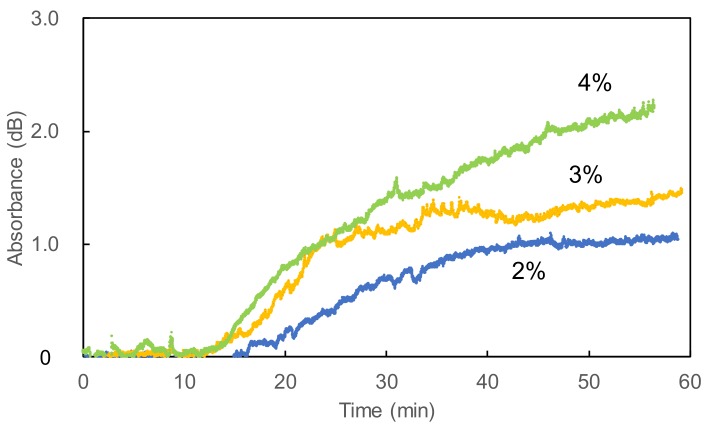
Temporal responses of CO_2_ concentrations in a sample bag measured using the optical fiber probe. The injection of CO_2_ gas was started at 10 min.

**Figure 9:**
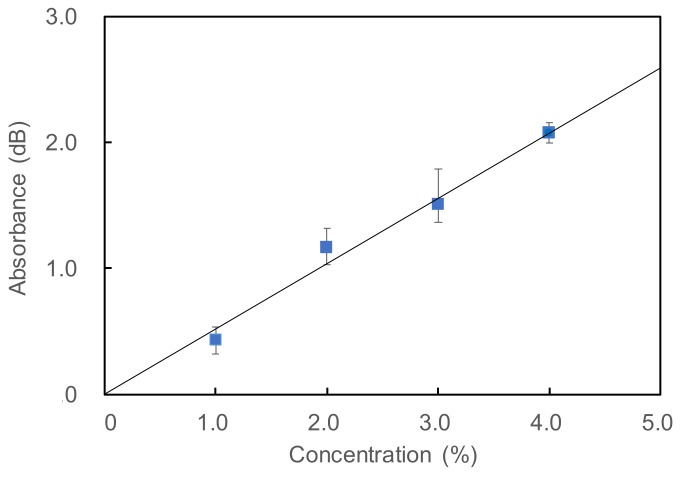
Calibration plot between measured optical absorbance and CO_2_ concentration.

Next, we measured the time responses of the system by injecting CO_2_ gases with different concentrations. Before each measurement, the background signal was obtained for the sample bag containing 100 mL of N_2_ gas. Then the sample gas was injected into the bag with a flow rate of 50 mL/min. Figure 8 shows temporal responses of optically measured CO_2_ concentrations where the injection of CO_2_ gas was started at 10 min. It was clear that the N_2_ gas in the bag was slowly purged with CO_2_ gas and the concentration increased with time. All the curves saturated after around 60 min except for the 4% concentration that saturated at around 80 min. Figure 9 shows a calibration plot for CO_2_ concentration. The measured absorbance was obtained from the saturated value shown in Figure 8, and each dot and error bar are averages and measurement errors of three respective measurements. The measurement errors were around ±0.15 dB, which corresponded to the concentration fluctuation of ±0.3%. We found that the fluctuation in the laser power was around 0.1 dB, and this suggested that the measurement errors in Figure 9 were mainly caused by the laser instability. Monitoring the laser power to correct obtained data may be effective to reduce the error, although it also causes a decrease in the obtained signal level. We confirmed that there was a highly linear correlation between the concentration and the optical absorbance. The correlation coefficient R^2^ was 0.981 and the repeatability in the experimental data was around 0.3%. Considering the measurement errors in the data for the 1.0% concentration, we concluded that the lowest detection limit for CO_2_ concentration was 0.45%.

**Figure 10:**
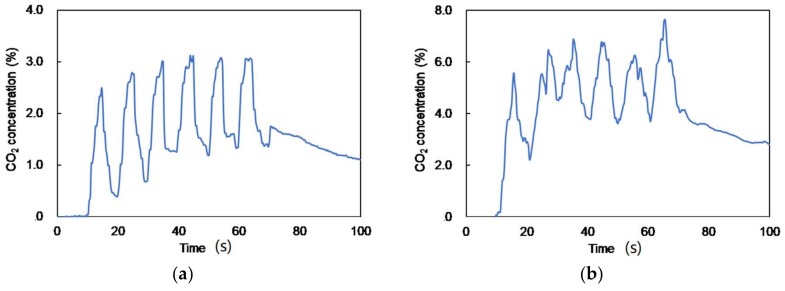
Results of real-time measurement of CO_2_ concentration in human breath: (**a**) measured at rest and (**b**) measured after exercise.

Figure 10a shows results of a real-time measurement of CO_2_ concentration in human breath. In this experiment, a healthy adult subject at rest breathed through a tube connected to a sample bag and the change in the CO_2_ concentration was measured using an optical fiber probe inserted into the bag. The CO_2_ concentration changed with the subject’s breaths in and out and was successfully measured by the optical absorption measurement. The baseline slowly drifted up because the gas in the sample bag was not totally replaced by each breath. In addition, we sometimes found that the mirror surface was clouded by moisture in breaths, which might have caused the drifts. Figure 10b shows a change in the CO_2_ concentration of human breath measured after the subject did some exercise (ran up the stairs to the fifth floor). We confirmed that the disordered breathing had a higher CO_2_ concentration than that measured at rest, as seen in Figure 10a.

**Figure 11:**
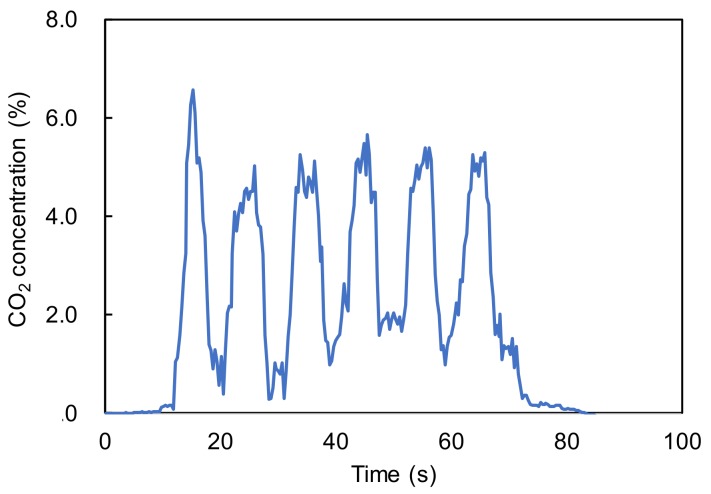
Change in the CO_2_ concentration measured inside a nostril.

Figure 11 shows a time response of CO_2_ concentration measured inside a nostril. In this experiment, a fiber probe was inserted into the nostril around 10 mm deep and changes in CO_2_ concentration was measured in real time. We confirmed that the changes in inhales and exhales were successfully measured, and the CO_2_ concentration measured was close to the typical value of 4% for human adults. This result showed that the baseline did not drift because the small volume of gas in the nostril was totally replaced by inspired and expired gases when breathing. This result confirmed the feasibility of our proposed fiber probe that can be inserted into human airways for real-time and in-situ measurement of CO_2_ concentration in human breaths.

## 4. Conclusions

For localized CO_2_ measurement in respiratory tracts, we proposed a real-time gas monitoring system based on optical absorption spectroscopy. In this system, a small gas cell was attached to the end of a hollow optical fiber that delivers mid-infrared light with a small transmission loss. The diameters of the fiber and the gas cell were smaller than 1.2 mm in order to insert the probe into a working channel of common bronchoscopes. First, the dimensions of the gas cell were designed based on absorption spectra of CO_2_ standard gases in the 4.2-μm wavelength region, which were measured using an FT-IR spectrometer. Then, a miniature gas cell comprised of a stainless-steel tube with slots for gas inlet and a micro-mirror was fabricated. A compact probing system with a QCL light source was built for monitoring CO_2_ concentration by using a gas cell with a hollow optical fiber.

The measurement error of the proposed system was ±0.3% and the minimum detection limit was 0.45% for CO_2_ concentration. Although these performances are inferior as compared to the other CO_2_ sensors, the sensor probe proposed in this paper is more robust, owing to the simple structure and the above performances are good enough to be used as an endoscopic CO_2_ sensor for respiratory tracts. The experimental results using human breaths show the feasibility of the system for in-situ measurement of localized CO_2_ concentration in human airways.
